# Self-efficacy and knowledge trial to improve sexual reproductive health and well-being for Syrian refugee women and girls in Lebanon: a study protocol for a randomized community-based trial

**DOI:** 10.3389/fgwh.2026.1793696

**Published:** 2026-08-06

**Authors:** Loulou Kobeissi, Sarah Ibrahim, Zahi Abdul Sater, Tania Bosqui, Gladys Honein-AbouHaidar, Theresa Farhat, Mohammad Fouad Mohammad Fouad, Hady Naal, Laura Hudroj, Dalia Sarieddine, Veloshnee Govender, Lale Say, Randa Hamadeh, Shadi Saleh

**Affiliations:** 1 Independent Consultant, Geneva, Switzerland; 2 Global Health Institute, American University of Beirut, Beirut, Lebanon; 3 Department of Psychology, American University of Beirut, Beirut, Lebanon; 4 School of Nursing, American University of Beirut, Beirut, Lebanon; 5 Liverpool School of Tropical Medicine, Liverpool, United Kingdom; 6 Department of Sexual and Reproductive Health, World Health Organization, Geneva, Switzerland; 7 Department of Primary Care, Ministry of Public Health, Beirut, Lebanon; 8 Faculty of Health Sciences, American University of Beirut, Beirut, Lebanon

**Keywords:** girls and young women, Lebanon, low resource intervention package, psychosocial support, randomized control trial, reproductive health, sexual health, Syrian refugees

## Abstract

**Background:**

Use and access to sexual and reproductive health (SRH) services are often inadequate and limited among adolescent girls and young refugee women in humanitarian settings. This is attributed to an array of factors including: limited health literacy, lack of knowledge on where and how to access services, limited availability of services, and gender norms. Further, the nature of humanitarian settings often poses additional mental health pressures because of limited safety, political instability, gender-based violence (GBV), and dire socio-economic and living conditions, among others. This is especially true for the Eastern Mediterranean Region (EMR), which hosts the largest number of humanitarian crises in the world, thus rendering the provision of SRH and psychosocial support (PSS) services in humanitarian settings highly compromised.

**Method:**

The proposed research aims to evaluate the impact of a WHO-developed low-intensity/low-resource psychosocial support (PSS) SRH-integrated intervention package on the use of selected SRH services, primarily family planning as well as well-being among Syrian adolescent girls and young women refugees, aged 15–24 years, in Lebanon. The research will adopt a community-based randomized controlled trial (RCT) design to evaluate the effectiveness of the PSS-SRH integrated intervention package on the use of selected SRH services as well as mental well-being. The RCT will be accompanied by a rigorous process evaluation during intervention implementation to assess intervention fidelity, attrition, dose and satisfaction, as well as to capture key lessons based on intervention implementation to inform global scale-up in different humanitarian settings.

**Discussion:**

This intervention is expected to be easily integrated into existing primary healthcare settings and specifically to improve selected SRH service use, leading to improved SRH outcomes and reduced SRH-related risks, and ultimately better wellbeing among refugee women and girls. Findings of this RCT are also expected to inform key stakeholders and better guide decision making for such interventions in humanitarian settings.

**Clinical Trial Registration**: https://clinicaltrials.gov/study/NCT07008950?term=self%20efficacy%20and%20knowledge&viewType=Card&rank=2, identifier: NCT07008950.

## Background

The Syrian crisis is considered one of the worst humanitarian emergencies in modern history (1). Since 2011, the crisis has led to over 13.4 million Syrian refugees in need for humanitarian assistance; more than 5.6 million of these refugees are hosted in countries near Syria, half of whom are women and girls of reproductive age (2). UNHCR estimates that over 1.5 million people from Syria currently reside in Lebanon, with only approximately 839,086 of them being UN-registered refugees. The remaining are unregistered individuals who are unable to return to Syria. Syrian refugees now amount to almost a quarter of the resident national Lebanese population, making Lebanon a country with the largest number of refugees per capita worldwide (3). Twelve years into this protracted crisis, Lebanon continues to struggle to keep pace, with no signs of any upcoming relief (3). This crisis has resulted in major public health challenges in Lebanon, including a significant strain on Sexual Reproductive Health (SRH) and mental health services for host communities and Syrian refugees (4). In addition to this crisis, Lebanon has been facing a compounded socioeconomic (Inform severity 3.6) and health crisis since 2019 (3), affecting all its resident population, including Lebanese citizens, refugees, and migrants, among others, with a disproportionate impact on the most vulnerable communities.

SRH problems are a leading cause of morbidity and mortality among girls and young women globally. During conflict or humanitarian emergencies, SRH knowledge and service use are often compromised and further compounded by increased SRH needs, violence, breakdown in health infrastructure, mental health difficulties and other vulnerabilities (5–8).

Evidence in Lebanon indicates an increased risk of sexual and gender-based violence (GBV), reproductive tract infections (RTIs), and pregnancy complications among Syrian refugee women (9, 10), all of which have been associated with increased morbidity and mortality (11). Rates of GBV are reported to exceed 30% among Syrian refugees (7–10) which further interferes with women's mobility and SRH choices and decisions (12). Access to family planning, which is critical for preventing unwanted pregnancies, unsafe abortion, and reducing maternal and child mortality, is reported to have decreased to 34.5% among Syrian women refugees living in Lebanon compared to 58.3% in Syria before the war (10). The same pattern is also observed for access to antenatal care. According to a study among Syrian refugees in Lebanon, a significant proportion of Syrian refugee women (up to 82.9%) reported only one antenatal visit while very few (only 15.7%) reported having four or more visits (13). A high prevalence of reproductive morbidities has also been reported among Syrian refugee women living in Lebanon, including menstrual irregularities (54%), pelvic pain (52%), and RTIs (53%) (10). The prevalence of MH problems was also found to be quite high among Syrian refugees in Lebanon, where it is estimated that 44% suffer from major depressive disorders, disproportionately affecting women (14), 38% from schizophrenia (15), and 27% from post-traumatic stress disorder (16). The high prevalence of MH problems is concerning as it interferes with the ability to make informed SRH decisions, often leading to inconsistent contraceptive use, increased risks of unwanted pregnancies, unsafe abortions and increased risk of human immunodeficiency virus (HIV) and other sexually transmitted infections (STIs) (17).

The aforementioned concerns have been equally voiced by the Ministry of Public Health (MoPH) and humanitarian agencies in Lebanon (4). The highest reported problems among Syrian refugee women include limited awareness of safe motherhood practices, symptoms of complications during pregnancy, and family planning. The main SRH stakeholders in the country agree that effective community outreach is needed to enhance early detection, timely referral, as well as responsive psychosocial support and counselling services among different Non-Governmental Organizations (NGOs) and aid agencies (4). Reported barriers to accessing the needed health services include limited availability of resources and infrastructure (18), geographical barriers, cost (19), and lack of adequate awareness and education about these issues and corresponding available services (4).

Against this backdrop, a WHO psychosocial support (PSS)—SRH integrated draft intervention package was developed to be delivered to Syrian refugee adolescent girls and young women in Lebanon. The draft intervention package consists of 8 modules focused on SRH education, psychoeducation, and enhancing skills acquisition. The draft intervention package is a low-resource/low intensity intervention, that can therefore be safely delivered by non-specialists following robust training.

This paper outlines the protocol for a Randomized Control Trial (RCT) that will be conducted in Lebanon to assess the effectiveness of the PSS-SRH draft intervention package.

## Methods

### Design

This study will employ a single-blinded community-based participatory research (CBPR) approach to assess the effectiveness of an integrated PSS-SRH intervention on Syrian adolescent girls and young women refugees (aged 15–24 years) residing in vulnerable communities in the Beqaa governorate in Lebanon. The trial consists of two arms: the experimental group (EG) who will receive the PSS-SRH intervention, and the control group (CG) who will receive the same educational material by the end of the study. The primary and secondary study outcomes will be measured at baseline (T0), end-line/post intervention (T1), and at 3-months post-intervention (T2). A comprehensive process evaluation, using a mixed-method study design, will also be conducted alongside the primary intervention outcome evaluation.

### Research aims and hypotheses

The overarching aim of this RCT is to assess the impact of a low-resource/low-intensity PSS-SRH integrated draft intervention package, augmented to existent health systems, on (1) the use of selected SRH services (specifically contraception/family planning use), and (2) the well-being of Syrian adolescent girls and young women refugees, by enhancing their self-efficacy and knowledge on said topics. This would include improving contraception/family planning use and well-being among the EG compared to the CG at 3 months-post intervention (T2).

This research study further aims to conduct a process evaluation to assess intervention fidelity and attrition during intervention implementation, as well as stakeholder satisfaction and key lessons learnt to inform global-scale up in different humanitarian settings.

### Study setting

The study will be implemented in two primary healthcare centers (PHCs) operating in the Beqaa governorate in Lebanon that will be selected based on recommendations from the Ministry of Public Health in Lebanon, department of primary healthcare. Both PHCs are required to meet the following criteria (1) originally offer SRH services to female Syrian refugees, (2) have well-kept medical records of beneficiaries, (3) able to offer indoor spaces for the implementation of the study, (4) have a large number of beneficiaries to meet the required sample size, (5) able to refer refugees with potential health problems (STIs, Mental Health, etc) to relevant health centers with adequate follow up capacity. The Beqaa governorate was selected for the purpose of the study as it hosts the highest number of registered refugees in Lebanon. The Beqaa accounts for 39% of the total registered Syrian refugee population as of January 2023 according to UNHCR, and is characterized by limited human resources and infrastructure. This allows for a comprehensive assessment of the impact of the draft intervention package on a population facing considerable challenges accessing adequate healthcare services.

### Study population

The target population of the study are Syrian adolescent girls and young women refugees (age 15–24 years) residing in Beqaa governorate in Lebanon. Participants will be included in the study if they meet the following inclusion criteria: (1) are currently married and between the ages of 15–24 years, (2) completed an average stay in Lebanon for a minimum of 6 months, (3) are willing to take part in the study as indicated by them signing an informed consent (for young women 18 years of age or older) or through a legal guardian consent form and written assent for adolescents' girls between 15 and 17, (4) have none of the exclusion criteria listed below upon screening.

Participants will be excluded from the study if they meet any of the following criteria: (1) are currently pregnant and/or lactating, (2) have reported chronic health problems interfering with the ability to follow the intervention protocol, (3) have high levels of anxiety and/or depression as well as suicidal ideations and/or attempts upon screening (based on the Hopkins 25) and/or a current diagnosis of a severe mental illness, or undergoing mental health treatment (20).

These criteria were intentionally selected to ensure delivery of the intervention to the intended target group. Particularly, enrolment will be restricted to currently married Syrian refugees because sexual health topics are considered sensitive and taboo subjects in the targeted community that would not be easily discussed with girls and young women who are not married due to cultural restrictions. Therefore, selecting this as an inclusion criterion reflects the intervention's focus on FP and SRH-related decision making among those most likely to require these services. Similarly, pregnant or lactating women and individuals with severe mental illness, may have needs or unique circumstances that may impact their ability to adequately adhere to the intervention schedule and therefore may benefit from other types of referrals or support. While it is acknowledged that these criteria may limit the generalisability of the findings, they are necessary to ensure participant safety and fidelity to the intervention.

### Sample size

Sample size calculation was based on a 20% improvement in the primary outcome (use of family planning) in the EG compared to CG at 3 months post intervention (T2). A 30% baseline prevalence of family planning use was assumed in the study population based on observed prevalence of family planning use in humanitarian settings, ranging from 7.1% (in Northern Uganda), 25% (among Afghan female refugees in Pakistan), and 34.5% (among Syrian women refugees in Lebanon) (9, 21–23). Allowing for 30% attrition, a sample size of 380 adolescent girls and young women would provide a 90% statistical power to detect a difference of 20% in family planning use at 3 months (from 30% to 50%) using a two-sided 5% significance level. This is equivalent to a relative risk of 1.67. To ensure external validity of the findings, a purposive sampling strategy will be employed, as the Syrian refugee population is not homogenous in Lebanon. Specifically purposive sampling will be used at the level of the site and community outreach, and not at the level of individual participant. Ultimately, the sample will be randomised from chosen PHCs that were selected to be part of this intervention based on specific criteria discussed in an earlier section.

### Recruitment

Recruitment will be conducted through a CBPR approach. It will be facilitated by the study stakeholders, including (1) the selected PHCs, (2) Ministry of Public Health (MoPH), (3) local women committee (LWC), and (4) community coalition (CC). LWCs are women refugees from the same target community who are facing similar, social, economic and familial hardship as the target population who have pre-existing or acquired, relationship and trust with them. This will strengthen participation, trust, and comfort of the target population in the research. The CC represents individuals perceived as trusted leaders [lay community members and representatives (community gatekeepers/directors of PHCs/reproductive health program managers, representatives from the Lebanese MoPH, NGOs, practitioners, health care providers, local Syrian refugee young women and so on)].

To preliminarily identify potentially eligible adolescent girls and young women, the research team in coordination with the selected PHCs will use the study eligibility criteria of age group and residence in Lebanon for a minimum of 6 months. The centers will then contact potentially eligible participants over the phone (if ≥18 years old) or their legal guardian(s) (if <18 years old) and invite them—using a pre-written invitation script**—**to participate in a series of health days. Health days will enable the research team to identify, select, and recruit eligible adolescent girls and young women to take part in the RCT following informed consent.

**Figure 1:**
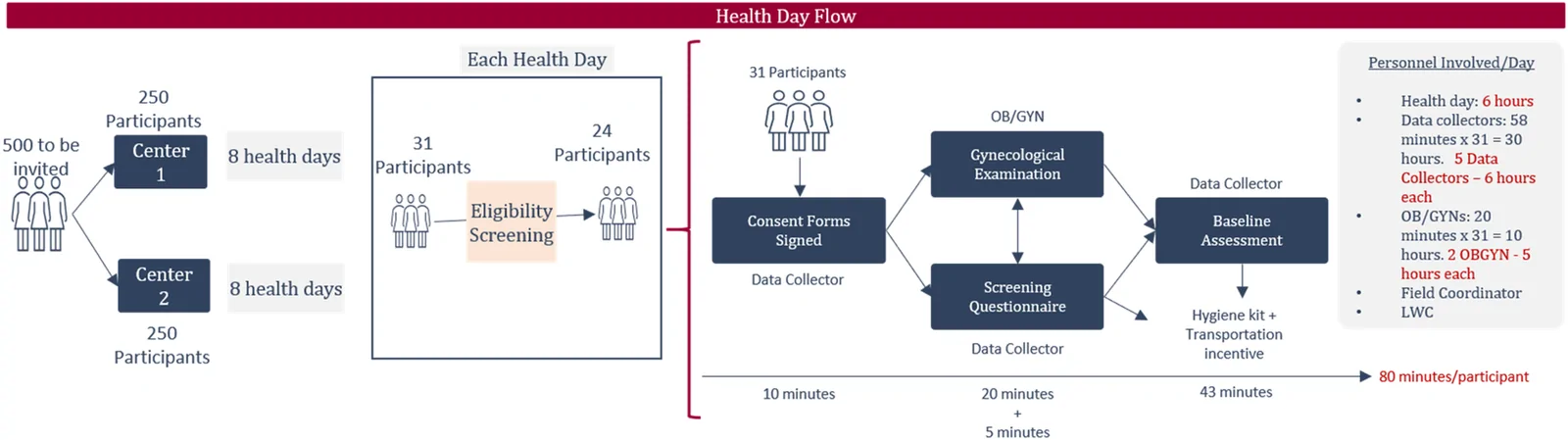
Recruitment schematic diagram.

Twelve to 20 health days are estimated to take place to recruit the target sample size (380 adolescent girls and young women). During the health days, the research team will explain the aims of the event and related activities in particular, and the RCT in general and will ask the invitees that are interested in participating in the study to sign a consent form or assent/parental consent. Those who consent to participate in the health days will be offered free SRH services, including an optional routine non-invasive Ob/GYN consultation, as well as women's hygiene kits and transportation compensation. Additionally, upon signing the consent form, potentially eligible participants will fill out a screening questionnaire to determine eligibility to participate in the study. If eligible, participants will fill out the baseline outcome evaluation questionnaire. If during the Ob/GYN exam, the physician recommends lab tests to rule out selected STIs and/or other morbidities, the participant will be referred to health facilities (MOPH PHCs) that offer these tests, along with future needed follow-ups for free. If potential participants are not eligible due to high levels of anxiety or depression, or suicidality identified during screening, they will be referred to free mental health services available in the region. The recruitment process is illustrated in Figure 1.

### Informed consent and assent

Consent forms will be inclusive of all research activities under the study. Two main types of consent forms will be administered depending on whether the participant is an adult (18 years or older) or an adolescent (aged 15–17 years). For adolescents, a written consent will be obtained from the legal guardian accompanying them in addition to written assent from the adolescent girl. To avoid undue influence, the team will first obtain assent from adolescent girls. Only if they agree to participate in the study will parental consent then be sought.

For all age groups, the field researcher will be accompanied by a member of the LWC to obtain consent and ensure that the potential participant is at ease.

To ensure respect of participants' autonomy, the team will emphasize voluntary participation and no coercion or undue influence will take place when taking consent. This includes reassuring participants that refusal or acceptance to participate would not in any way influence the benefits and services they already receive from the PHC or other organizations. Issues of confidentially and willingness to leave the research at any time, even after consent, will be highlighted as well.

Although the intervention does not inherently pose any risks of harm to participants, emotional distress may surface as a result of the topics addressed by some participants. Because this is a vulnerable population, several steps will be considered to minimise undue influence on them. This includes ensuring the presence of psychologists and midwives in each session to support with emotional distress and other challenges that may arise during the session, but also systems for referral within the PHC or to other NGOs that provide specialised care in relation to protection, health, and others. Participants will be encouraged to share their experiences with the professionals and facilitators and with their peers, and the intervention will be delivered in a manner that is scientific and that encourages learning.

### Randomization

Randomization will be conducted after baseline (T0) assessments are completed and will be stratified by site. Eligible participants will be randomly allocated to EG (intervention group) or CG with a 1:1 ratio. Randomization will be conducting using a computer-generated allocation schema, generated by the study statistician. The randomization schema will use permuted block design with sizes of 4, 6, and 8 blocks in a random mixture, so that treatment allocations are equal after each block size. The young women and adolescents will then be informed of their allocation into either the EG or CG over the telephone.

Participants in the EG will be further divided into subgroups of no more than 12 participants per group. These smaller subgroups facilitate better engagement, interaction, and personalized attention, promoting a supportive and comfortable environment for the participants.

### Intervention

**Figure 2:**
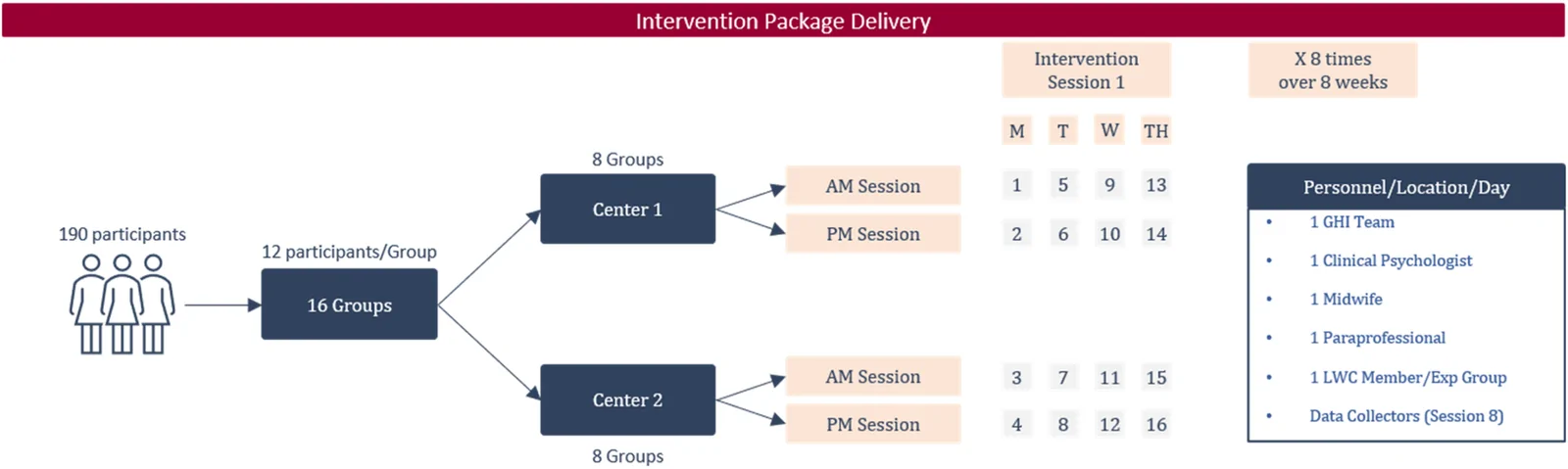
Intervention package delivery schematic diagram.

The WHO PSS-SRH draft intervention package consists of eight modules/sessions focused on SRH education, psychoeducation, and enhancing skills acquisition (specifically around: emotional regulation, communication, problem management, decision-making, and self-efficacy). It is designed to be delivered by trained community paraprofessionals (non-specialists) with at least twelve years of formal education. The draft intervention package will be administered (at the selected PHCs) once a week (for each subgroup) for a duration of 90 min over a total of 8 consecutive weeks. To create a supportive and collaborative environment for the participants in the study, each LWC member will be assigned to an EG subgroup and will attend all their sessions. The involvement of LWC members is expected to build trust and ensure a culturally sensitive approach throughout the intervention delivery. This will play a crucial role in fostering a sense of community among participants, encouraging open discussions, and facilitating the sharing of experiences and ideas. A midwife and a clinical psychologist will also attend all sessions of the intervention to support the paraprofessional when/if needed and to ensure protocol adherence as well as quality and content fidelity. Importantly, the psychologist and midwife will also support participants during the session should they feel distressed, especially when discussing sensitive topics. In such an event, the psychologist or midwife would first attend to the concerns of the participant separately, and if the need arises, they may refer them to relevant care services from professionals within the given PHC or to specialised care outside of the PHC. The intervention delivery schematic (which includes personnel, location, and days of intervention delivery) is displayed in Figure 2.

### Facilitator selection and training

A team of women paraprofessionals (non-specialists), from the target population, will be responsible for implementing the intervention package. The paraprofessionals will be selected, with the help of the PHCs, LWC, and CC, based on the criterion of having a minimum of 12 years of formal education. These paraprofessionals will receive a twenty-day comprehensive training facilitated by a clinical psychologist and a SRH expert, both of whom have experience working with Syrian refugee populations. The training will equip them with the necessary skills and knowledge to effectively deliver the intervention content in a standardized manner and address any concerns or questions that may arise during the sessions. The training will cover a range of topics, including ethical training, session implementation, role playing, and strategies for managing challenging scenarios. To ensure their readiness to implement the intervention, the paraprofessionals performance will be assessed by the team during the training, who will also select the top performing paraprofessionals to participate in the intervention delivery.

### Ethical approval

This RCT has received ethical approval from the Institutional Review Board (IRB) at the American University of Beirut (AUB) (SBS-2023-0119 and SBS-2023-0310) and from World Health Organization (WHO) Research Project Review Panel (RP2) (A66058.5865) and Ethical Review Committee (ERC), both of which are in accordance with the Declaration of Helsinki.

### Blinding

A single-blinded design was chosen because study participants will be aware of whether they are in the experimental or control group, since they would either receive or not receive the intervention package. The team collecting the data on the other hand will not be aware of which participants are in the control and which are in the experimental groups, since post-intervention questionnaires do not inquire about participants' receipt of the intervention. Additionally, data collectors will be explicitly instructed not to inquire about this information when in contact with participants.

### Contamination

Contamination risks are considered minimal in this intervention. Primarily, this is because the sessions will focus on skill building and is largely participatory through in-class group discussions. This means that it is unlikely for learnings to be transmitted to others who are not part of the experimental group. Participants will also be asked not to share any information about the intervention with others to maintain confidentiality and privacy of the group. They will also be asked not to share material or knowledge with others until the end of the study period. Because contamination risks were deemed minimal as this intervention focuses on skill building and is highly participatory, potential contamination will not be formally measured. This may present a limitation that should be considered when interpreting findings.

### Outcome measures

To thoroughly evaluate the impact and effectiveness of the intervention package, we will employ a comprehensive set of questionnaires (incorporating various tools) that will be administered to both the EG and CG. These questionnaires will enable the research team to thoroughly assess the impact of the intervention package on various aspects of the participants' lives, including their well-being and SRH. Refer to Table 1 for a detailed description of each tool and the time of administration.

**Table 1:** Outcome evaluation tools and time of administration.

Outcome	Aim	Tool	Study period			
			Screening	Baseline T0	End-line T1 (8 weeks)	3 months post-intervention T2 (20 weeks)
Participant Inclusion	Inclusion and exclusion	Screening tool (24 questions)	X			
Primary outcome	At least 20% improvement in family planning use among the EG compared to the CG at three months.	Pan Arab-Family Health (PAPFAM) survey (24) (BL: 68 questions; EL: 35 questions; P3M: 37 questions)		X	X	X
Secondary outcomes	Improved well-being among the EG compared to the CG at three months. Multiple dimensions of well-being will be assessed.	Hopkins Symptom Checklist 25 (25 questions) (20, 25, 26)		X	X	X
		WHO-5 Wellbeing (5 questions) (27)		X	X	X
		General Self-Efficacy Scale (10 questions) (28)		X	X	X
		Multidimensional Scale of Perceived Social Support for Arab Women (12 questions) (29)		X	X	X
Intermediate outcomes	Improvement in important mediators in the EG compared to the CG	The Difficulties in Emotion Regulation Scale Short Form DERS-SF (18 question) (30, 31)		X	X	X
		Interpersonal Communication Competency scale (32) (17 question)		X	X	X
		Revised Ways of Coping Checklist (12 questions) (33)		X	X	X
Important variables and confounder	Such as socio-demographic factors; Family planning including other SRH knowledge; Women's reproductive and obstetric history; Gender disadvantage; and previous history of domestic violence	Using questions from the PAPFAM Survey (24).		X	X	X

Outcome evaluation will be conducted at baseline (T0) followed by the implementation of the intervention. The end-line outcome evaluation (T1) will be conducted on the final session (8th session) of the intervention. Subsequently, the post-3 months outcome evaluation (T2) will be conducted 3 months after T1. In cases where the participants do not attend a scheduled assessment (T1 or T2), three attempts will be made in the same week, followed by one in the subsequent week to schedule a new appointment via telephone.

The majority of the tools have been used and validated in Arabic in various settings. One of the tools, Interpersonal Competency Scale (ICCS) was not available in Arabic and was therefore translated into simple, colloquial Arabic that can be understood by participants (i.e., low literacy participants) following recommended procedures for cross-cultural research. Translation followed the WHO Guidelines on Translation. The steps included: forward translation to Arabic by a translator (who is a bilingual, native Arabic speaker) with a psychological/health background. Next, an expert panel, which consists of 2–3 bilingual experts in the field, was held to identify and resolve the inadequate expressions/concepts of the translation, as well as any discrepancies between the forward translation and the original document. After that, the back translation to English was be done by a translator who is also a bilingual, native English speaker. The last step will was pre-testing the translated version with a population similar to the target population (15–24 year old female Syrian refugees). This stage included cognitive interviews asking the participants what they think of each translated component, which was done through interviews.

Outcome evaluation instruments will be administered through a single questionnaire, in person, on a tablet using an electronic data collection software, KoBo. All data collected through KoBo will be stored on password-protected tablets and secured on institutional servers the American University of Beirut. Access to study data will be authorised only to members of the research team with password protected accounts. Data shared with study statistician will be anonymised and participants will be identified by codes without any personal information assigned to them. Should adverse events be identified during data collection, management, or analysis, the protocol states that they be immediately directed towards the study's principle investigator to determine the appropriate course of action. The study will employ female data collectors as some women may feel more at ease in discussing sensitive topics related to SRH with individuals of the same gender. Prior to their enrolment in the study, the data collectors will receive a training on various aspects, including the different sections of the questionnaires, research ethics, risk of bias in collecting data, reporting procedures of adverse events, data management, managing distress, obtaining informed consent, as well as referral mechanisms. The training, which will span over two days, will provide the data collectors the opportunity to engage in role-playing to ensure proper data collection methods.

To ensure that the research questionnaire is easy to understand and accurately captures the necessary data, a pilot will be conducted with a group of Syrian refugees similar to the target population before the actual initiation of the RCT. The purpose of the pilot test is to identify survey issues such as unclear wording or confusing questions or the length of the outcome evaluation questionnaire. Feedback collected from the pilot test will be used to refine the questionnaire for use in the actual RCT.

#### Primary outcome

The primary outcome of the intervention is to improve contraception/family planning use among the EG compared to the CG at 3 months. This will be measured by the Pan Arab-Family Health (PAPFAM) survey (34). The PAPFAM survey is similar to the well-known Demographic and Health Survey in design but with a focus on family health in the Arab countries. It uses a standardized instrument to collect data mainly on maternal health and reproductive health of women as well as data on child, adolescent, and elderly health. The study will use only relevant sections of the PAPFAM survey, chosen based on their alignment with the study's objectives and research questions. Specifically, questions that assess participants' knowledge, attitudes, and practices related to family planning methods, and utilization of family planning services have been extracted. Other questions relating to socio-economic status, marriage, and STI knowledge have also been extracted.

#### Secondary outcomes

The secondary objective of the intervention is to improve mental well-being among the EG compared to the CG at 3 months. To measure the improvement in mental well-being, changes in common mental distress, quality of life, self-efficacy, and social support will be assessed.

Common mental distress will be measured by the Hopkins Symptoms Checklist-25 (HSCL-25) (20). The HSCL-25 will be used to measure the participants' level of depression, anxiety, and stress.

Quality of life will be measured by the World Health Organization Well-Being 5-item Index (WHO-5) (27). The WHO-5 is a short 5-item instrument that measures psychological well-being, including depression. Each of the 5 positively worded items is rated on a 6-point Likert scale ranging from 0 (constantly present or all the time) to 5 (not present or at no time). The summed scores range from 0 to 25. A score <13 indicates poor wellbeing or low mood, though not necessarily depression and warrants further assessment (35).

Self-efficacy will be measured using the General Self-Efficacy Scale (GSES) (28). It is a 10-items scale designed to assess a person's optimistic self-beliefs used to cope with life's demands.

Social support will be measured by the Multidimensional Scale of Perceived Social Support for Arab Women (MSPSS-AW) (29). The 12- Item tool will assess the participants' perceptions of the availability and quality of emotional, instrumental, and informational support from friends, family, and community members.

#### Intermediate outcomes

The intermediate outcomes will measure improvement in important mediators in the EG compared to CG at three months. Mediators include emotional regulation, communication skills, and coping, problem management and decision making.

Emotional regulation will be measured by the Difficulties in Emotion Regulation Scale Short Form (DERS-SF). The DERS-SF consists of 18-items, with participants rating their answers in the past week on a scale from 1 (almost never) to 5 (almost always). Total scores range from 18 to 90, with higher scores indicating more difficulty in emotional regulation (34).

Communication skills will be measured by the Interpersonal Communication Competency Scale (ICCS). The tool assesses ten dimensions of interpersonal communication competence (self-disclosure, empathy, social relaxation, assertiveness, alter-centrism, interaction management, expressiveness, supportiveness, immediacy, and environmental control. A total of 17 questions will be selected from the scale based on a study done in Brazil (32).

Coping, strategies, problem solving, and decision making will be assessed using the Revised Ways of Coping Checklist (RWCCL), which measures different types of coping will also be used. It will enable the research team to evaluate the structure of the interpersonal or individual social supports as well as their collective or networked social supports. The RWCCL is a widely used multidimensional coping measure with satisfactory reliability and validity (33, 36). Specific subscales were chosen based on our aim, and to avoid overlapping questions. The included subscales are: Confrontive coping, Escape-Avoidance, and Planful problem-solving.

### Analysis

A detailed statistical analysis plan will be developed upon completion of the study. The data will first be downloaded from the electronic data collection software, KoBo, and imported into a statistical analysis software.

An Intent-to-treat analysis approach will be adopted to test for significant difference of primary and secondary outcomes over time. For baseline (T0) comparability, descriptive statistics (frequency distributions, means, standard deviations, medians, IQRs) will be obtained for variables collected at baseline outcome evaluation. The Chi-square test and t-test will be used to investigate whether potentially confounding variables are balanced across the two study groups. Any potential bias due to these potential confounders will be evaluated analytically by considering them in the regression model. The internal and external study validity will be evaluated by comparing baseline characteristics of those who completed the study to those lost to follow-up, and by comparing screening characteristics of those included in the study to those eligible but not consented for inclusion in the study.

For primary outcome analysis, binary outcome of FP use (yes/no) will be evaluated between baseline (T0) and endline (T1), and between baseline and 3-month follow-up (T2). To estimate treatment effects, multivariable logistic regression models will be conducted separately for T0-T1 and T0-T2 comparisons to estimate odds ratios of FP use in the intervention group compared to the control group. All models will be adjusted for potential sociodemographic confounders. Given that each model will include one change outcome per participant, within-participant correlation as a result of repeated measures will not arise within individual models.

The primary analysis will be repeated 1- to adjust for potential confounders identified in the bivariate analysis, 2- in the compliant “per protocol” subgroup that attended 6 out of 8 (75%) weekly sessions, 3- to explore the impact of missing data with multiple imputation methods, and 4- to consider the change in FP use from intervention completion to three months.

Analysis of secondary outcomes will be conducted at endline (T1) and 3 months post-intervention (T2) 1- to evaluate the potential increase in the secondary outcomes (well-being), and 2- in the use of the other SRH services associated with the intervention; 3- to investigate potential mediators of the primary comparison; 4- to investigate the effect of co-interventions and adherence. Mediators will be assessed as additional explanatory variables to the primary logistic regression models.

## Discussion

Improving SRH and PSS knowledge coupled with skills-building around SRH self-efficacy is expected to improve selected SRH service use, mainly family planning use, which could ultimately lead to better SRH outcomes such as averting unplanned pregnancies, reducing complications of unsafe abortion, pregnancy, childbirth, and STIs among adolescent girls and young women refugees. Additionally, commensurate improvements in the secondary outcome, well-being, is also expected (37).

The developed intervention package is designed to be easily integrated into existing health systems, such as in routine PHC or social services settings at the national level, specifically in humanitarian settings effectively, efficiently, and in a culturally appropriate manner. This is in line with the WHO building block #1, as it could improve access and use of essential health services that are often compromised in humanitarian and fragile settings. The integration of SRH-PSS services at times of urgency and need is critical, given the scarcity of resources and the stringent abilities for continued and sustained health care for refugees and displaced populations in humanitarian settings (38).

In addition, it could lead to improved quality of care, as the integration of health services could yield more efficient use of health care resources, including human resources, community health care workers, and other paraprofessionals to deliver such service, in line with the WHO building block #2: empowering the health care workforce.

Finally, the findings of this research will provide potentially useful insights for the adaptation of the intervention package for young people in humanitarian contexts. This is essential in informing decisions of key stakeholders for broader dissemination and scale-up in humanitarian settings. The findings may also be useful for public health policymakers, as they could broaden the understanding of what works more effectively and for whom in fragile contexts.
